# Supplementary figures and images for: PrimedSherlock: a tool for rapid design of highly specific CRISPR-Cas12 crRNAs
Source: BMC Bioinformatics. 2022 Oct 14;23:428. doi: 10.1186/s12859-022-04968-5 (PMC9569017; doi:10.1186/s12859-022-04968-5)

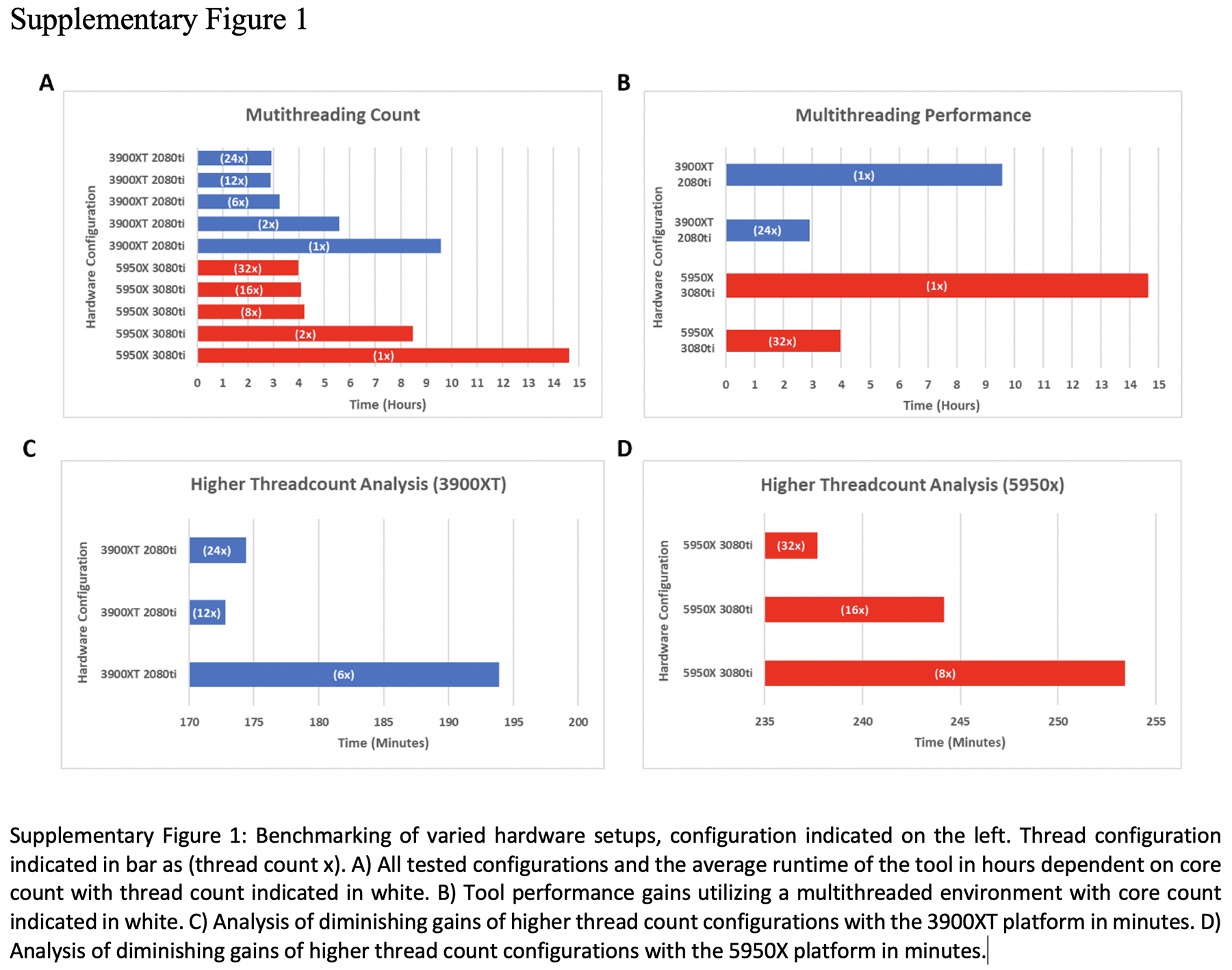

Supplement: Supplementary file 1 — Additional file 1. Figure S1. Benchmarking of varied hardware setups, configuration indicated on the left. Thread configuration indicated in bar as (thread count x). A) All tested configurations and the average runtime of the tool in hours dependent on core count with thread count indicated in white. B) Tool performance gains utilizing a multithreaded environment with core count indicated in white. C) Analysis of diminishing gains of higher thread count configurations with the 3900XT platform in minutes. D) Analysis of diminishing gains of higher thread count configurations with the 5950X platform in minutes. [file 12859_2022_4968_MOESM1_ESM.tiff]
